# An observational study of the effectiveness and safety of nivolumab plus chemotherapy for untreated advanced or recurrent gastric cancer in Japanese real-world settings: the G-KNIGHT study

**DOI:** 10.1007/s10120-025-01641-7

**Published:** 2025-07-09

**Authors:** Shigenori Kadowaki, Tomoyuki Otsuka, Keiko Minashi, Shinichi Nishina, Hiroshi Yabusaki, Chiaki Inagaki, Tomohiro Nishina, Hisateru Yasui, Hiroshi Matsuoka, Nozomu Machida, Masahiro Tsuda, Fumio Nagashima, Hisashi Hosaka, Junichi Matsubara, Hiroyuki Arai, Satoshi Ida, Yuya Kimijima, Yuko Matsuda, Manabu Muto, Kei Muro

**Affiliations:** 1 Department of Clinical Oncology, Aichi Cancer Center Hospital, Aichi, Japan; 2 Department of Medical Oncology, Osaka International Cancer Institute, Osaka, Japan; 3 Clinical Trial Promotion Department, Chiba Cancer Center, Chiba, Japan; 4 Department of Medical Oncology, Kurashiki Central Hospital, Okayama, Japan; 5 Department of Gastroenterological Surgery, Niigata Cancer Center Hospital, Niigata, Japan; 6 Department of Medical Oncology, Faculty of Medicine, Kindai University, Osaka, Japan; 7 Department of Gastrointestinal Medical Oncology, NHO Shikoku Cancer Center, Ehime, Japan; 8 Department of Medical Oncology, Kobe City Medical Center General Hospital, Hyogo, Japan; 9 Department of Surgery, Fujita Health University, Aichi, Japan; 10 Department of Gastroenterology, Kanagawa Cancer Center, Kanagawa, Japan; 11 Department of Gastroenterological Oncology, Hyogo Cancer Center, Hyogo, Japan; 12 Department of Medical Oncology, Faculty of Medicine, Kyorin University, Tokyo, Japan; 13 Department of Gastroenterology, Gunma Prefectural Cancer Center, Gunma, Japan; 14 Department of Medical Oncology, Kyoto University Graduate School of Medicine, Kyoto, Japan; 15 Department of Clinical Oncology, St. Marianna University School of Medicine, Kanagawa, Japan; 16 Department of Gastroenterological Surgery, Graduate School of Medical Sciences, Kumamoto University, Kumamoto, Japan; 17 Oncology Medical, Bristol Myers Squibb, Tokyo, Japan; 18 Medical Affairs, Ono Pharmaceutical Co. Ltd, Osaka, Japan

**Keywords:** Japan, Nivolumab, Observational study, Stomach neoplasms

## Abstract

**Background:**

Nivolumab plus chemotherapy has shown efficacy in clinical trials for advanced or recurrent gastric cancer (GC). However, real-world utilization data are limited. In this study, we aimed to assess the effectiveness, safety, and treatment status of first line nivolumab plus chemotherapy in Japanese patients with treatment-naïve advanced or recurrent GC.

**Methods:**

Untreated patients with advanced or recurrent GC who initiated nivolumab plus chemotherapy as first line treatment from November 2021 to June 2023 across 23 Japanese sites were enrolled in this observational study (G-KNIGHT). This report focused on the objective response rate (ORR), real-world progression-free survival (rwPFS), and the treatment-related adverse event (TRAE) incidence. Furthermore, subgroup analyses for ORR and rwPFS were conducted for patients stratified by various factors including age and the programmed cell death ligand 1 (PD-L1) combined positive score (CPS).

**Results:**

Among 527 patients (median age, 70.3 years; 25.2% aged ≥ 75 years; 65.5% male; 84.3% with advanced GC), the median follow-up period was 10.4 (interquartile range, 6.7–15.2) months. The ORR was 65.6% (95% confidence interval [CI], 59.9–70.9%). The median rwPFS (months, 95% CI) was 6.9 (6.2–7.6); by subgroups: age < 75 years, 6.7 (6.0–7.5); age ≥ 75 years, 7.4 (6.2–8.6); PD-L1 CPS < 1, 7.5 (6.5–9.0); CPS 1–5, 6.2 (5.5–8.0); and CPS ≥ 5, 7.0 (6.2–8.2). TRAEs occurred in 91.3% of patients, with 40.4% experiencing grade ≥ 3 events.

**Conclusions:**

This large-scale real-world study supports the effectiveness and safety of nivolumab plus chemotherapy in untreated Japanese patients with advanced or recurrent GC.

**Supplementary Information:**

The online version contains supplementary material available at 10.1007/s10120-025-01641-7.

## Background

Gastric cancer (GC) remains a significant global health burden, ranking as the fifth most common cancer [1]. It was the fifth leading cause of cancer-related deaths worldwide in 2020 [1], although mortality from GC is decreasing [2]. In Japan, GC was projected to account for an estimated 40,711 deaths (10.6% of all cancer deaths) in 2022 [3, 4], reflecting its continued impact on public health. In addition, patients with GC are progressively older in Japan, with the incidence rate in those aged ≥ 75 years exceeding 50% [4, 5]. This trend shows the importance of understanding treatment status data for older patients in Japan.

Conventional treatments, such as 5-fluorouracil, platinum, and taxane administration, have recently been used to manage unresectable advanced or recurrent GC. Although advances in chemotherapeutic agents have enabled notable tumor shrinkage, complete remission by pharmacological treatment remains elusive. The main goals of treating unresectable advanced or recurrent GC are to alleviate symptoms, delay disease progression, and extend patient survival. However, significant unmet medical needs remain in this area, particularly for older patients with reduced tolerance to chemotherapy and those with massive ascites [6].

The treatment paradigm for unresectable advanced or recurrent GC has evolved from conventional chemotherapy to targeted immunotherapies. Nivolumab, a programmed cell death-protein 1 (PD-1) inhibitor, was approved in Japan as third line monotherapy for GC in 2017, based on the results of the ATTRACTION-2 trial [7]. Nivolumab as first line treatment in combination with chemotherapy was approved in Japan after the CheckMate 649 [8] and ATTRACTION-4 [9] trials of its use in patients with human epidermal growth factor receptor 2 (HER2)-negative, treatment-naïve advanced or recurrent GC. The long-term results of the CheckMate 649 [10] and ATTRACTION-4 [11] trials also demonstrated the consistent efficacy of nivolumab plus chemotherapy. Nivolumab plus chemotherapy has been incorporated as a therapeutic option in clinical guidelines, including the National Cancer Care Network Gastric Cancer Guidelines [12], the Japanese Gastric Cancer Treatment Guidelines 2025 (7th edition) [6], and the European Society for Medical Oncology Clinical Practice Guideline [13]. In addition, with the new treatment approvals of zolbetuximab plus chemotherapy in 2024, based on the results of the SPOTLIGHT [14] and GLOW [15] trials, and pembrolizumab plus chemotherapy in 2024, based on the results of the KEYNOTE-859 trial [16], the first line treatment options for advanced and recurrent GC in Japan have expanded.

Despite these advances, the real-world effectiveness and safety profile of nivolumab plus chemotherapy remain to be fully elucidated. The CheckMate 649 and ATTRACTION-4 trials imposed partial restrictions on the inclusion of individuals with ascites and excluded those with an Eastern Cooperative Oncology Group performance status (ECOG PS) of ≥ 2 [8, 9]. Moreover, the recruited patients were relatively young [8, 9], which does not reflect the aging patient population in Japan. Therefore, obtaining data on the treatment of older patients in real-world settings is important. Furthermore, CheckMate 649 included a limited number of Japanese patients. Meanwhile, ATTRACTION-4 included a large number of Japanese patients; however, this study lacked stratified efficacy analyses according to the programmed cell death-ligand 1 (PD-L1) combined positive score (CPS) [9]. According to the Japanese Gastric Cancer Treatment Guidelines 2025 (7th edition) [6], systemic chemotherapy remains clinically questionable for patients with impaired oral intake or massive ascites as well as older patients with poor performance status. Moreover, while selection of first line treatment based on biomarkers, including PD-L1 CPS, is recommended in this guideline, data on treatment outcomes stratified by PD-L1 CPS specifically for Japanese patients remains limited.

The G-KNIGHT study aimed to assess the real-world effectiveness, safety profile, and actual treatment status of first line nivolumab plus chemotherapy for treatment-naïve advanced or recurrent GC.

## Methods

### Study design

The G-KNIGHT study is an ongoing observational study that assesses the effectiveness, safety, and conditions of treatment of patients with treatment-naïve, advanced, or recurrent GC with first line nivolumab plus chemotherapy in a real-world setting at 23 participating sites in Japan (Online Resource 1). As prespecified in the protocol for four sequential investigations, this analysis was based on the second planned investigation, collecting data through November 2023. This analysis focused on real-world progression-free survival (rwPFS), the objective response rate (ORR), and early safety outcomes. Final data collection will continue through December 2025.

Patients with treatment-naïve advanced or recurrent GC who received nivolumab plus chemotherapy as first line treatment from November 2021 to June 2023 were enrolled. CyberOncology® is a system provided by the Prime Research Institute for Medical RWD, Inc. (Kyoto, Japan), which standardizes and structures drug and disease information obtained in clinical practice, such as electronic medical record data, to compile a database. The system enables the standardized collection of oncology data across institutions. For this study, we configured it to capture protocol-specified variables from electronic medical records. Each patient’s treatment plan was determined at the physician’s discretion in accordance with the package insert and Guidelines for the Promotion of Optimal Use. Data collection proceeded after patients provided written informed consent. For patients unable to provide written consent themselves or through legal representatives, an opt-out approach was implemented for study enrollment in accordance with the Japanese Ethical Guidelines for Medical and Health Research Involving Human Subjects.

This study was registered with the UMIN Clinical Trials Registry (UMIN000046820) and ClinicalTrials.gov (NCT05334719).

### Patients

We enrolled patients who met the following criteria: (1) age ≥ 20 years; (2) histologically confirmed advanced or recurrent GC; (3) scheduled or initiated treatment with nivolumab and chemotherapy. Combination chemotherapy with S-1/oxaliplatin (SOX), capecitabine/oxaliplatin (CapeOX), or 5-fluorouracil/leucovorin/oxaliplatin (FOLFOX) was permitted. We excluded patients who received antineoplastic agents as first line treatment for advanced or recurrent GC prior to the initiation of nivolumab and chemotherapy. However, patients who received prior perioperative chemotherapy (neoadjuvant and/or adjuvant) or bisphosphonates for osseous metastases may still have been enrolled. The first line treatment status for such patients was determined in accordance with the clinical judgment of the treating physician, consistent with the real-world nature of this observational study. We also excluded patients who had confirmed HER2-positivity; had previously received investigational drugs with antitumor effects after a GC diagnosis; or had begun treatment with nivolumab plus chemotherapy as first line treatment for advanced or recurrent GC at a site other than the study site and were later hospitalized at the study site.

### Objectives

The primary, secondary, and exploratory objectives of the G-KNIGHT study are presented in Online Resource 2. In this analysis, we examined the ORR, duration of response (DOR), rwPFS, overall survival (OS), duration of treatment (DOT), and time to next treatment (TNT) of nivolumab plus chemotherapy, incidences of immune-related AEs (irAEs) and treatment-related AEs (TRAEs) leading to treatment discontinuation, time to onset and recovery of irAEs, and actual treatment status, including treatment sequence. In addition, subgroup analysis was performed according to patient characteristics and the association between patient characteristics and rwPFS.

### Assessment

The severity of AEs was assessed according to the Common Terminology Criteria for Adverse Events v5.0. AE information, such as whether the event was an irAE, its severity, its causal relationship with the treatment, and its outcome, was assessed by the investigators. Regarding to AE outcome, recovery included recovered, recovering, and recovered but with sequelae. Radiological tumor evaluation was performed by each investigator according to the Response Evaluation Criteria in Solid Tumors v1.1 (RECIST) methodology, using images obtained in clinical practice. Progression disease was determined not only by radiological progression based on RECIST v1.1, but also by clinical progression, which was assessed by worsening of disease status based on clinical symptoms, physical findings, tumor markers, and various test values. Histopathological classification of GC was based on the pathological diagnosis made at each study site according to the Japanese Classification of Gastric Carcinoma 15th edition [17]. Evaluations of PD-L1 CPS (immunohistochemistry 28–8), microsatellite instability (MSI), HER2, and laboratory test results were based on the results obtained at each study site. If PD-L1 CPS was not evaluated at the individual study sites, we performed a central test at SRL, Inc. (Tokyo, Japan) on the stored specimens, if available.

### Statistical analysis

The target sample size was set at 500 patients based on the feasibility, because this is an observational study without formal statistical hypothesis testing.

All analyses were performed using a population of consenting patients, after excluding the ineligible patients. Data are presented as medians and ranges or interquartile ranges for continuous variables, and as numbers (percentage) for categorical variables. The treatment duration was measured in two ways: (1) drug-specific duration from initiation to discontinuation of each medication, and (2) DOT from first line treatment initiation to the physician-determined end date of the treatment. For patients who received multiple regimens, the drug-specific approach categorized them in each respective treatment group, while the DOT approach categorized them only once according to their regimen with the longest duration. The Kaplan–Meier method was used to estimate the median DOR, rwPFS, and OS, with 95% confidence intervals (CIs) calculated using the Brookmeyer–Crowley method. The ORR was calculated with 95% CIs using the Clopper–Pearson method. Hazard ratios (HRs) for time-to-event endpoints with 95% CIs were calculated using Cox proportional hazards regression. Multivariable logistic regression analysis was performed to explore patient characteristics and laboratory tests associated with disease progression and with the duration of nivolumab plus chemotherapy. Variables included in the multivariable model were selected according to their clinical relevance, statistical significance in the univariable analysis (cutoff, *p* < 0.05), and correlation between variables. The details of the analytical method are described in Online Resource 3. Statistical analyses were conducted using SAS v. 9.4 (SAS Institute Inc., Cary, NC, USA) and R v4.2.2 (https://www.r-project.org/; R Software for Statistical Computing, Vienna, Austria) for drawing the swimmer plot.

## Results

### Patients and treatment

A total of 539 patients were enrolled in this study; 527 patients with HER2-negative GC who received nivolumab plus chemotherapy as first line treatment were included in this analysis, excluding those who did not meet the eligibility criteria (Online Resource 4). The median follow-up period was 10.4 (interquartile range, 6.7–15.2) months. Details regarding the patient characteristics are described in Table 1. The median age of the included patients was 70.3 (range, 24–87) years. Of the included patients, 133 (25.2%) were aged ≥ 75 years; 345 (65.5%) were male; and 255 (48.4%), 224 (42.5%), and 36 (6.8%) had an ECOG PS of 0, 1, and ≥ 2, respectively. Regarding histology, 188 patients (35.7%) had an undifferentiated tumor type while 317 (60.2%) had a differentiated tumor type. Lymph node metastasis was the most common (291 patients; 55.2%), followed by peritoneal dissemination (243 patients; 46.1%) and liver metastasis (130 patients; 24.7%). PD-L1 CPS was tested in 451 patients (85.6%); 89 (19.7% of the 451 patients), 143 (31.7%), and 216 patients (47.9%) had tumors with a CPS of < 1, 1–5, and ≥ 5, respectively. MSI was tested in 220 patients (41.7%); 5.9% (13 of the 220 patients) had MSI-high tumors. Overall, 204 patients (38.7%) presented with ascites. Among the patients with ascites, 157 patients (77.0%) had mild-to-moderate ascites, 23 patients (11.3%) had massive ascites, and 24 patients (11.8%) had unclear results. Patient characteristics according to the PD-L1 CPS and combination chemotherapy regimens are shown in Online Resource 5.

**Table 1:** Patient characteristics

Characteristics, Overall n = 527	n (%)
Age, years	
Median (range)	70.3 (24–87)
< 65	172 (32.6)
≥ 65, < 75	222 (42.1)
≥ 75	133 (25.2)
Male	345 (65.5)
ECOG PS	
0	255 (48.4)
1	224 (42.5)
2	32 (6.1)
3	3 (0.6)
4	1 (0.2)
Unknown	12 (2.3)
Primary tumor	
Gastric cancer	456 (86.5)
Gastroesophageal junction cancer	71 (13.5)
Histology	
Undifferentiated	188 (35.7)
Differentiated	317 (60.2)
Others	22 (4.2)
Disease status	
Advanced	444 (84.3)
Recurrent or relapse	83 (15.7)
Metastasis	
Lymph node	291 (55.2)
Peritoneal dissemination	243 (46.1)
Liver	130 (24.7)
Bones and joints	40 (7.6)
Bronchi and lungs	25 (4.7)
Brain, brainstem, spinal cord, ventricles, and cerebellum	2 (0.4)
Stomach	1 (0.2)
Others	53 (10.1)
No. of metastasis	
0	40 (7.6)
1	261 (49.5)
≥ 2	226 (42.9)
PD-L1 CPS IHC 28–8	
Tested	451 (85.6)
< 1	89/451 (19.7)
≥ 1	359/451 (79.6)
1–5	143/451 (31.7)
< 5	232/451 (51.4)
≥ 5	216/451 (47.9)
Unclear	3/451 (0.7)
Untested	37 (7.0)
Unknown	39 (7.4)
MSI status	
Tested	220 (41.7)
MSS	192/220 (87.3)
MSI-low	3/220 (1.4)
MSI-high	13/220 (5.9)
Unclear	12/220 (5.5)
Untested	166 (31.5)
Unknown	141 (26.8)
HER2 IHC	
IHC 0	294 (55.8)
IHC 1 +	167 (31.7)
IHC 2 + and ISH-	66 (12.5)
IHC 3 +	0
Ascites	
Without	271 (51.4)
With	204 (38.7)
Mild to moderate	157/204 (77.0)
Massive	23/204 (11.3)
Unclear	24/204 (11.8)
Unknown	52 (9.9)
Adjuvant chemotherapy	
Without	455 (86.3)
With	67 (12.7)
Unknown	5 (0.9)
Previous surgery	139 (26.4)
Laboratory tests	
Albumin, g/dL	
n (%)	521 (98.9)
Mean ± SD	3.4 ± 0.6
Lactate dehydrogenase, U/L	
n (%)	517 (98.1)
Mean ± SD	250.4 ± 276.1
C-reactive protein, mg/dL	
n (%)	511 (97.0)
Mean ± SD	1.7 ± 2.9
White blood cell, count/mm^3^	
n (%)	520 (98.7)
Mean ± SD	7,190.1 ± 2,998.7
Hemoglobin, g/dL	
n (%)	520 (98.7)
Mean ± SD	11.5 ± 1.9
Neutrophil, count/mm^3^	
n (%)	351 (66.6)
Mean ± SD	5,072.0 ± 2,723.1
Alkaline phosphatase, U/L	
n (%)	521 (98.9)
Mean ± SD	136.8 ± 150.7

Abbreviations: CPS, combined positive score; ECOG PS, Eastern Cooperative Oncology Group performance status; HER2, human epidermal growth factor receptor 2; IHC, immunohistochemistry; ISH, in situ hybridization; MSI, microsatellite instability; MSS, microsatellite stability; PD-L1, programmed cell death ligand 1; SD, standard deviation

At the data cutoff point, 114 patients (21.6%) continued to receive nivolumab plus chemotherapy. The most common reason for treatment discontinuation was ineffectiveness (n = 282, 53.5%) followed by AEs (n = 63, 12.0%) and patient requests (n = 9, 1.7%) (Table 2). The median duration of nivolumab treatment was 3.5 (range, 0.0–26.1) months, reflecting the drug-specific duration from initiation to discontinuation of nivolumab. The combination regimens for nivolumab plus chemotherapy were primarily SOX (n = 393, 74.6%), CapeOX (n = 36, 6.8%), and FOLFOX (n = 109, 20.7%), with 11 patients treated with multiple regimens. The median DOT—representing the overall first line treatment duration from initiation to the physician-determined end date—was 6.3 (95% CI, 5.8–7.0) months in patients with nivolumab plus SOX, 8.2 (95% CI, 4.4–18.0) months in those treated with nivolumab plus CapeOX, and 5.0 (95% CI, 3.8–6.5) months in those treated with nivolumab plus FOLFOX (Online Resource 6). Overall, 53 patients (10.1%) underwent surgery after nivolumab plus chemotherapy initiation.

**Table 2:** Treatment status

Parameter	Overall n = 527	NIVO + SOX* n = 393, 74.6%	NIVO + CapeOX* n = 36, 6.8%	NIVO + FOLFOX* n = 109, 20.7%
Treatment status, n (%)				
On-going	114 (21.6)	87 (22.1)	12 (33.3)	17 (15.6)
Discontinued	413 (78.4)	306 (77.9)	24 (66.7)	92 (84.4)
Discontinued reason, n (%)				
Ineffective	282 (53.5)	213 (54.2)	14 (38.9)	63 (57.8)
Adverse event	63 (12.0)	49 (12.5)	5 (13.9)	9 (8.3)
Patient requests	9 (1.7)	8 (2.0)	0	1 (0.9)
Death	8 (1.5)	3 (0.8)	2 (5.6)	3 (2.8)
Other reasons	49 (9.3)	32 (8.1)	3 (8.3)	15 (13.8)
Unknown	2 (0.4)	1 (0.3)	0	1 (0.9)
NIVO duration^†^, n (%)				
Median (range), months	3.5 (0.0–26.1)	3.5 (0.0–26.1)	3.7 (0.0–24.8)	3.4 (0.0–17.0)
< 6 months	367 (69.6)	270 (68.7)	22 (61.1)	79 (72.5)
≥ 6–12 months	113 (21.4)	88 (22.4)	8 (22.2)	22 (20.2)
≥ 12–18 months	37 (7.0)	26 (6.6)	5 (13.9)	8 (7.3)
≥ 18–24 months	7 (1.3)	7 (1.8)	0	0
≥ 24 months	3 (0.6)	2 (0.5)	1 (2.8)	0
Fluoropyrimidine duration^†^				
Median (range), months	3.6 (0.0–26.0)	3.7 (0.0–26.0)	3.7 (0.0–24.9)	3.0 (0.0–17.0)
Oxaliplatin duration^†^				
Median (range), months	2.9 (0.0–19.6)	2.9 (0.0–19.6)	3.1 (0.0–11.3)	3.0 (0.0–8.3)

^*^ A total of 11 patients were treated with multiple regimens, and they were categorized in each regimen

^†^ Drug duration was calculated as the period from the initiation date to the discontinuation date of each medication, based on prescription data extracted from electronic medical records

Abbreviations: CapeOX, capecitabine/oxaliplatin; FOLFOX, 5-fluorouracil/leucovorin/oxaliplatin; NIVO, nivolumab; SOX, S-1/oxaliplatin

### Effectiveness

**Fig. 1:**
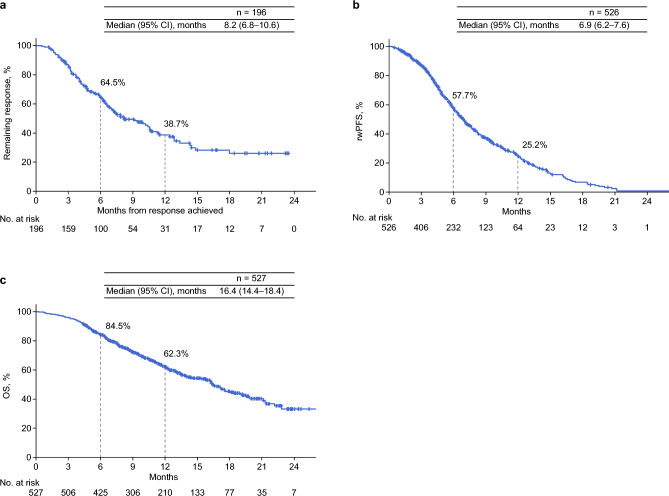
Effectiveness outcomes **a** DOR, **b** rwPFS*, and **c** OS *One patient without available data on the date of confirmed rwPFS after nivolumab initiation was excluded from the analysis. Abbreviations: CI, confidence interval; DOR, duration of response; OS, overall survival; rwPFS, real-world progression-free survival

In total, 527 patients were evaluated for effectiveness. Among 299 patients with measurable diseases and response evaluation, the ORR and disease control rate were 65.6% (95% CI, 59.9–70.9%) and 93.0% (95% CI, 89.5–95.6%), respectively, with eight (2.7%) patients achieving a complete response, 188 (62.9%) achieving a partial response, and 82 patients (27.4%) achieving stable disease (Table 3). Among patients with an objective response, 64.5% did not show progression within 6 months, with a median duration remaining response of 8.2 (95% CI, 6.8–10.6) months (Fig. 1a).

**Table 3:** ORR

Parameter, Overall* n = 299	
BOR, n (%)	
CR	8 (2.7)
PR	188 (62.9)
SD	82 (27.4)
PD	21 (7.0)
ORR, (%)	65.6
95% CI	59.9–70.9
DCR, (%)	93.0
95% CI	89.5–95.6

^*^ Data were analyzed in 299 patients with measurable disease and response evaluation, which were assessed by investigators per Response Evaluation Criteria in Solid Tumors v1.1. The denominator of BOR, ORR, and DCR is the number of patients with measurable disease and response evaluation. Abbreviations: BOR, best overall response; CI, confidence interval; CR, complete response; DCR, disease control rate; ORR, objective response rate; PD, progressive disease; PR, partial response; SD, stable disease

The median rwPFS was 6.9 (95% CI, 6.2–7.6) months, with a 6-month rwPFS rate of 57.7% (Fig. 1b). The median OS was 16.4 (95% CI, 14.4–18.4) months, with 6-month and 12-month OS rates of 84.5% and 62.3%, respectively (Fig. 1c).

**Fig. 2:**
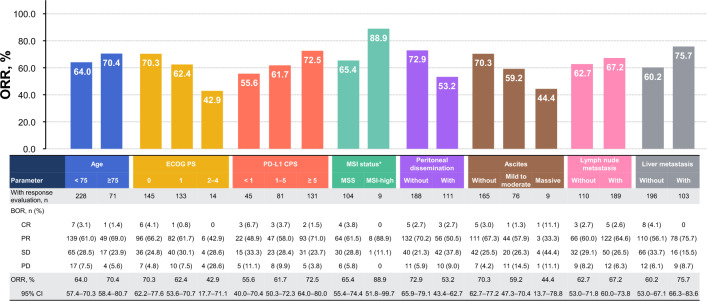
ORR by subgroup ORR was analyzed in patients with measurable disease and response evaluation, which were assessed by investigators per Response Evaluation Criteria in Solid Tumors v1.1. *MSI-low is not shown because only one patient was applicable. Abbreviations: BOR, best overall response; CI, confidence interval; CPS, combined positive score; CR, complete response; DCR, disease control rate; MSI, microsatellite instability; MSS, microsatellite stability; ORR, objective response rate; PD, progressive disease; PD-L1, programmed cell death ligand 1; PR, partial response; SD, stable disease

**Fig. 3:**
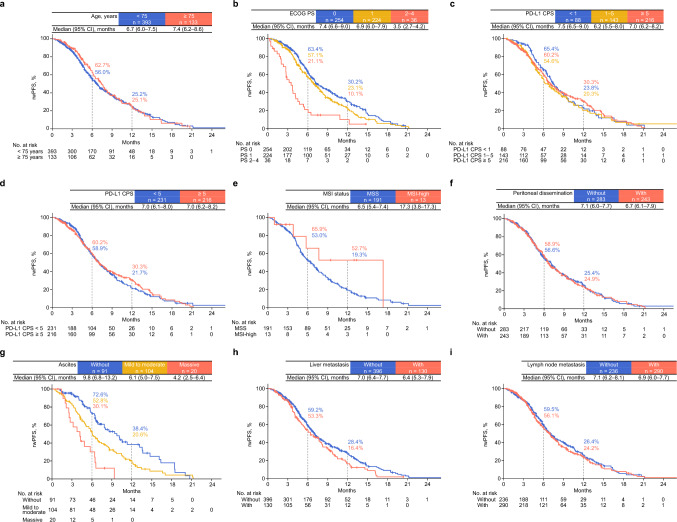
rwPFS* by subgroups **a** Age (< 75 and ≥ 75 years), **b** ECOG PS (0, 1, and ≥ 2), **c** PD-L1 CPS^†^ (< 1, 1–5, and ≥ 5), **d** PD-L1 CPS^†^ (< 5 and ≥ 5), **e** MSI status^‡^ (MSS and MSI-H), **f** peritoneal dissemination (without and with), **g** ascites in patients with peritoneal dissemination^§^ (without, mild to moderate, and massive), **h** liver metastasis (without and with), and **i** lymph node metastasis (without and with). *One patient without available data on the date of confirmed rwPFS after nivolumab initiation was excluded from the analysis. ^†^Seventy-nine patients without available PD-L1 CPS data were excluded from the analysis. ^‡^A total of 319 patients without available MSI data were excluded from the analysis. MSI-L is not shown because only 3 patients were applicable. ^§^Twenty-eight patients with ascites whose ascites severity was unknown were excluded from the analysis. Abbreviations: CI, confidence interval; ECOG PS, Eastern Cooperative Oncology Group performance status; CPS, combined positive score; MSI, microsatellite instability; MSS, microsatellite stability; PD-L1, programmed cell death ligand 1; rwPFS, real-world progression-free survival; MSI, microsatellite instability

Figures 2 and 3 show the results of subgroup analyses of ORR and rwPFS. By age, the ORR and median rwPFS were 64.0% (95% CI, 57.4–70.3%) and 6.7 months (95% CI, 6.0–7.5) for patients aged < 75 years and 70.4% (95% CI, 58.4–80.7%) and 7.4 months (95% CI, 6.2–8.6) for those aged ≥ 75 years, respectively (Figs. 2 and 3a). According to ECOG PS, the ORR was 70.3% (95% CI, 62.2–77.6%) for ECOG PS 0, 62.4% (95% CI, 53.6–70.7%) for ECOG PS 1, and 42.9% (95% CI, 17.7–71.1%) for ECOG PS ≥ 2 (Fig. 2), while the median rwPFS was 7.4 months (95% CI, 6.6–9.0), 6.9 months (95% CI, 6.0–7.9), and 3.5 months (95% CI, 2.7–4.2) for PS 0, 1, and ≥ 2, respectively (Fig. 3b). Based on PD-L1 CPS, the ORR and median rwPFS were 55.6% (95% CI, 40.0–70.4%) and 7.5 months (95% CI, 6.5–9.0), 61.7% (95% CI, 50.3–72.3%) and 6.2 months (95% CI, 5.5–8.0), and 72.5% (95% CI, 64.0–80.0%) and 7.0 months (95% CI, 6.2–8.2) for CPS < 1, 1–5, and ≥ 5, respectively (Figs. 2 and 3c). In patients without and with peritoneal dissemination, the ORRs were 72.9% (95% CI, 65.9–79.1%) and 53.2% (95% CI, 43.4–62.7%), respectively; the corresponding median rwPFS durations were 7.1 months (95% CI, 6.0–7.7) and 6.7 months (95% CI, 6.1–7.9), respectively (Fig. 3f). Regarding ascites, the ORR was 70.3% (95% CI, 62.7–77.2%) in patients without ascites, 59.2% (95% CI, 47.3–70.4%) in those with mild to moderate ascites, and 44.4% (95% CI, 13.7–78.8%) in those with massive ascites (Fig. 2). Among patients with peritoneal dissemination, the median rwPFS was 9.8 months (95% CI, 6.8–13.2) for those without ascites, 6.1 months (95% CI, 5.0–7.5) for mild to moderate ascites, and 4.2 months (95% CI, 2.5–6.4) for massive ascites (Fig. 3g). ORR and rwPFS data for MSI status, lymph node metastasis, and liver metastasis were also analyzed and are presented in Figs. 2 and 3.

### Second line treatment

Among the 413 patients who discontinued nivolumab plus chemotherapy, 196 (47.5%) received second line treatment (Online Resource 7). The most common second line treatment regimen was paclitaxel plus ramucirumab (n = 79, 40.3%) or ramucirumab plus nab-paclitaxel (n = 79, 40.3%). The median TNT was 6.7 (range, 0.7–21.0) months (Online Resource 7).

### Association of patient characteristics with rwPFS

Online Resource 8 summarizes the results of univariable and multivariable analyses of rwPFS. After multivariable analysis, ECOG PS (0–1 vs. 2–4; HR, 2.04; 95% CI, 1.23–3.38; *p* = 0.006), ascites (without vs. mild to moderate; HR, 1.26; 95% CI, 0.94–1.70; without vs. massive; HR, 2.79; 95% CI, 1.60–4.86; *p* = 0.001), and albumin per 1 g/dL increase (HR, 0.76; 95% CI, 0.61–0.96; *p* = 0.020) remained as significant factors affecting rwPFS.

### Safety

Any grade and grade 3–4 TRAEs were observed in 481 and 213 patients (91.3% and 40.4%), respectively, with 55 patients (10.4%) discontinuing treatment due to TRAEs (Table 4). Any grade and grade 3–4 irAEs were observed in 136and 41 patients (25.8% and 7.8%), respectively, with the most common being endocrine disorders (59 patients [11.2%]) among irAEs of any grade. Treatment-related deaths occurred in five patients (0.9%). Pneumonitis caused death in four patients (0.8%), while febrile neutropenia caused the death in one patient (0.2%). Among the five patients with treatment-related death, three were aged < 75 years and two were aged ≥ 75 years; one had ECOG PS 0, three had ECOG PS 1, and one had unknown PS. A summary of safety by subgroup of patients by age and ECOG PS scores is presented in Online Resource 9.

**Table 4:** Safety summary

	NIVO + Chemo, n = 527	
Category	Any grade, n (%)	Grade 3–4, n (%)
TRAE	481 (91.3)	213 (40.4)
TRAE leading to discontinuation	55 (10.4)	27 (5.1)
irAE	136 (25.8)	41 (7.8)
Endocrine	59 (11.2)	11 (2.1)
Skin	28 (5.3)	3 (0.6)
Pulmonary	24 (4.6)	7 (1.3)
Gastrointestinal	22 (4.2)	7 (1.3)
Hepatic	14 (2.7)	9 (1.7)
Renal	1 (0.2)	1 (0.2)
Others	19 (3.6)	9 (1.7)

The severity of AEs was assessed according to Common Terminology Criteria for Adverse Events v5.0 by the investigators. AE information, such as whether the event was an irAE, its severity, and its causal relationship with the treatment, and its outcome, was assessed by the investigators. Treatment-related death was observed in five patients (0.9%). Pneumonitis caused the death of four patients (0.8%). Febrile neutropenia caused the death of one patient (0.2%). Abbreviations: AE, adverse event; NIVO + Chemo, nivolumab plus chemotherapy; irAE, immune-related adverse event; TRAE, treatment-related adverse event

The median time from nivolumab plus chemotherapy initiation to the onset of any grade irAE was 3.7 months (range, 0–24.6 months; Online Resource 10). The median time from nivolumab plus chemotherapy initiation to onset of grade ≥ 3 irAEs was 3.0 (range, 0.4–24.6) months (Online Resource 11). Of the 199 any grade irAEs, 135 (67.8%) resolved, with a median time from onset to recovery of 5.0 (range, 0.7–27.2) months (Online Resource 12). Of the 57 grade ≥ 3 irAEs, 42 (73.7%) resolved, a median time from onset to recovery of 5.1 (range, 1.8–27.2 months) months (Online Resource 13).

## Discussion

This is the first report on the effectiveness, safety, and actual treatment status of first-line nivolumab plus chemotherapy in patients with untreated, advanced, or recurrent GC/gastroesophageal junction cancer (GEJC), based on multicenter, large-scale, real-world data from Japan. A key strength of this study was the inclusion of patient populations often underrepresented in clinical trials, such as those with poor ECOG PS (≥ 2) or massive ascites—groups for whom systemic chemotherapy is often considered clinically questionable [6].

Despite their inclusion, the clinical outcomes, including ORR, rwPFS, OS, and DOR, supported the results obtained in two clinical trials, CheckMate 649 and ATTRACTION-4 [8, 9]. In addition, the safety profile of nivolumab plus chemotherapy in our study was consistent with that obtained in the clinical trials, and no new safety signals were identified. Indeed, our study offers several points for discussion regarding these unresolved clinical questions highlighted in current Japanese guidelines [6], particularly in terms of outcomes for older patients, those undergoing biomarker-driven treatment selection, and patients with peritoneal dissemination and massive ascites.

Given that optimal treatment strategies for older patients are highlighted as a key clinical question in current Japanese guidelines [6], combined with the pressing need for more specific real-world data, this study distinctively featured a higher proportion of patients aged ≥ 75 years, unlike CheckMate 649 and ATTRACTION-4 which recruited relatively younger patients [8, 9]. This is crucial for addressing the clinical challenges posed by Japan's rapidly aging demographic [4, 5] and providing crucial real-world data for this group. In patients aged ≥ 75 years and those aged < 75 years, a particularly meaningful observation was the comparable effectiveness of the treatment and the numerically similar incidence rates of TRAEs and irAEs. This suggests the potential benefits and acceptable toxicity of nivolumab plus chemotherapy in older populations.

In terms of biomarker-driven treatment selection, various treatment options, including recently approved zolbetuximab (for claudin-18 isoform 2 [CLDN18.2]-positive tumors) and pembrolizumab, are available for unresectable advanced or recurrent GC; thus, appropriate treatment selection is important to ensure patient benefits. To support treatment selection the Japan Gastric Cancer Association and the Pan-Asia European Society for Medical Oncology Clinical Practice Guidelines recommend simultaneous testing for four biomarkers: HER-2, CLDN18.2, PD-L1, and MSI/mismatch repair [18, 19]. Treatment selection should be guided by the results of biomarker tests. In this study, among the four guideline-recommended biomarker, we focused on patients who were HER2 negative and performed subgroup analyses of effectiveness by PD-L1 CPS and MSI status. While there was a trend toward differential median PFS and ORR across PD-L1 CPS subgroups in CheckMate 649 [8], our real-world study showed consistent rwPFS across the PD-L1 CPS subgroups. However, ORRs were consistent with the trend observed in a previous Japanese study [20]. This finding diverges from the expectation that a higher PD-L1 CPS would correlate with better PFS. Interestingly, we observed no substantial differences in patient backgrounds across the PD-L1 CPS subgroups. Our real-world definition of progression included clinical deterioration without radiological confirmation, introducing an element of subjectivity when compared with RECIST-based assessments in controlled trials. In addition, we could not prespecify the frequency of imaging evaluations for the detection of disease progression, and the assessments were based on examinations with methodology and frequency decided at the discretion of the physician. These methodological factors may be attributed to the absence of notable differences in rwPFS by PD-L1 CPS, different from the findings in some controlled trials with standardized assessments. As demonstrated in our study, the value of PD-L1 CPS as a predictive biomarker for nivolumab plus chemotherapy in real-world clinical settings should be discussed further. In terms of MSI, the proportion of patients with MSI-high was similar in this study (5.9%) and CheckMate 649 (3%) [8] and WJOG13320GPS (5.6%) [21]. Regarding efficacy, CheckMate 649 demonstrated that the median OS was 38.7 months for patients with MSI-high and 13.8 months for patients with microsatellite stability (MSS) [22], indicating a particularly pronounced survival benefit for patients with MSI-high. Although we did not evaluate OS for patients with MSI-high in our study, the ORR was 88.9% for MSI-high and 65.4% for MSS. The median rwPFS was 17.3 months for patients with MSI-high and 6.5 months for patients with MSS; this indicated favorable outcomes for patients with MSI-high. The outcomes of OS analysis in future long-term follow-up studies are eagerly awaited. Thus, caution is necessary when using PD-L1 CPS as a biomarker, as opposed to the use of MSI, which is a reliable biomarker, even though it pertains to a smaller population. Our findings suggest that integration of biomarker results with the drug profiles, and the patient’s preferences are crucial, while also taking the patient’s clinical background into account.

On another front, while effective therapeutic strategies for patients with peritoneal dissemination and massive ascites remain a key clinical question, our study offers pertinent findings in this area. In ATTRACTION-4, the median PFS was 7.46 months for patients with peritoneal metastasis and 15.21 months for those without peritoneal metastasis [9]. In ATTRACTION-2 [23] which is later third line nivolumab monotherapy, the presence of peritoneal dissemination was associated with shorter PFS. Our real-world study further refined this observation among patients with peritoneal dissemination, showing that there was no difference in rwPFS based on the presence or absence of peritoneal dissemination. However, among patients with peritoneal dissemination, those without ascites demonstrated a longer median rwPFS than those with ascites. Consistent with this finding, our multivariable analysis of rwPFS in patients with advanced or recurrent GC treated with nivolumab plus chemotherapy identified the presence of ascites, but not the presence of peritoneal dissemination, as an independent factor associated with poor outcomes, along with ECOG PS 2–4 and decreased albumin levels. These factors were similar to those previously reported [24–26], resulting in understandable outcomes. To date, several clinical trials have focused on patients with peritoneal metastasis and ascites. In studies examining the use of FOLFOX as first-line therapy for gastric cancer with severe peritoneal metastasis, the median PFS has been reported to be 7.5 months [27]. Of the patients who received nivolumab plus FOLFOX in the present study, 61.3% had peritoneal dissemination and 56.6% had ascites. Regarding treatment duration, the DOT was numerically shorter in patients receiving FOLFOX than in those receiving other regimens, this may have been influenced by the fact that the FOLFOX regimen group in this real-world setting included more patients with poor ECOG PS and severe ascites (Online Resource 5). Our observations suggest that nivolumab might not provide additional benefits for patients with severe peritoneal metastasis. Hence, this finding warrants further investigation through dedicated prospective studies.

Our study had several limitations. First, the observational design and short-term follow-up may have limited the generalizability and causal inference. Given the short follow-up duration, the current survival outcomes should be viewed as preliminary observations that require confirmation through longer-term data analyses. The planned final analysis with extended follow-up will provide more robust OS data, enabling more comprehensive assessment of long-term outcomes. In addition, the absence of a control group further constrained our ability to draw definitive conclusions regarding treatment effect. Second, incomplete patient enrollment and reliance on electronic medical records may have introduced selection bias and missing data, potentially affecting the representativeness of our sample. Third, the lack of standardized imaging evaluation frequency across sites may have introduced variability in the assessment of disease progression. Finally, our site selection process, which was efficient for patient recruitment, may have limited the broader applicability of our results to all clinical settings. The participating sites were selected from among high-volume centers across Japan that had substantial experience in treating advanced or recurrent GC and had either already implemented CyberOncology® or were willing to adopt it for the study. While this approach facilitated systematic and efficient data collection, it may have introduced selection bias toward larger or more technologically advanced institutions, which potentially limits the generalizability of our findings in Japanese real-world settings. Despite these limitations, our study's use of electronic medical records and structured data extraction through CyberOncology® enabled efficient data collection and rapid dissemination of real-world evidence. This approach allowed the systematic accumulation of treatment responses and AE data, providing valuable insights into the effectiveness and safety of nivolumab plus chemotherapy for advanced or recurrent GC in a real-world setting. Our findings provide valuable real-world evidence that complements data from randomized controlled trials. The large sample size and inclusion of diverse patient populations enhanced the generalizability of our results to clinical practice.

In conclusion, this large-scale study provided valuable insights into the use of nivolumab plus chemotherapy in untreated Japanese patients with advanced or recurrent GC/GEJC. It supported the effectiveness and safety of this regimen across various patient subgroups, including those involving older patients and those based on PD-L1 CPS, in a real-world setting.

## Supplementary Information

Below is the link to the electronic supplementary material.

Supplementary file1 (DOCX 292 KB)
