# Correction: An observational study of the effectiveness and safety of nivolumab plus chemotherapy for untreated advanced or recurrent gastric cancer in Japanese real-world settings: the G-KNIGHT study

**DOI:** 10.1007/s10120-026-01772-5

**Published:** 2026-06-18

**Authors:** Shigenori Kadowaki, Tomoyuki Otsuka, Keiko Minashi, Shinichi Nishina, Hiroshi Yabusaki, Chiaki Inagaki, Tomohiro Nishina, Hisateru Yasui, Hiroshi Matsuoka, Nozomu Machida, Masahiro Tsuda, Fumio Nagashima, Hisashi Hosaka, Junichi Matsubara, Hiroyuki Arai, Satoshi Ida, Yuya Kimijima, Yuko Matsuda, Manabu Muto, Kei Muro

**Affiliations:** 1 Department of Clinical Oncology, Aichi Cancer Center Hospital, Aichi, Japan; 2 Department of Medical Oncology, Osaka International Cancer Institute, Osaka, Japan; 3 Clinical Trial Promotion Department, Chiba Cancer Center, Chiba, Japan; 4 Department of Medical Oncology, Kurashiki Central Hospital, Okayama, Japan; 5 Department of Gastroenterological Surgery, Niigata Cancer Center Hospital, Niigata, Japan; 6 Department of Medical Oncology, Faculty of Medicine, Kindai University, Osaka, Japan; 7 Department of Gastrointestinal Medical Oncology, NHO Shikoku Cancer Center, Ehime, Japan; 8 Department of Medical Oncology, Kobe City Medical Center General Hospital, Hyogo, Japan; 9 Department of Surgery, Fujita Health University, Aichi, Japan; 10 Department of Gastroenterology, Kanagawa Cancer Center, Kanagawa, Japan; 11 Department of Gastroenterological Oncology, Hyogo Cancer Center, Hyogo, Japan; 12 Department of Medical Oncology, Faculty of Medicine, Kyorin University, Tokyo, Japan; 13 Department of Gastroenterology, Gunma Prefectural Cancer Center, Gunma, Japan; 14 Department of Medical Oncology, Kyoto University Graduate School of Medicine, Kyoto, Japan; 15 Department of Clinical Oncology, St. Marianna University School of Medicine, Kanagawa, Japan; 16 Department of Gastroenterological Surgery, Graduate School of Medical Sciences, Kumamoto University, Kumamoto, Japan; 17 Oncology Medical, Bristol Myers Squibb, Tokyo, Japan; 18 Medical Affairs, Ono Pharmaceutical Co. Ltd, Osaka, Japan

**Correction to: Gastric Cancer (2025) 28:955–967** **https://doi.org/10.1007/s10120-025-01641-7**

In the sentence beginning “Among 299 patients with measurable…” in this article should have read “Among 299 patients with measurable diseases and response evaluation, the ORR and disease control rate were 65.6% (95% CI, 59.9–70.9%) and 91.6% (95% CI, 87.9–94.5%), respectively, with eight (2.7%) patients achieving a complete response, 188 (62.9%) achieving a partial response, and 78 patients (26.1%) achieving stable disease (Table 3).”

In Table 3 and Fig. 2 of this article, the values for stable disease (SD), progressive disease (PD), and DCR were incorrect. The corrected Table 3 and Fig. 2 should read as given here.

**Table 3:** ORR

Parameter, Overall* n = 299	
BOR, n (%)	
CR	8 (2.7)
PR	188 (62.9)
SD	78 (26.1)
PD	25 (8.4)
ORR, (%)	65.6
95% CI	59.9–70.9
DCR, (%)	91.6
95% CI	87.9–94.5

* Data were analyzed in 299 patients with measurable disease and response evaluation, which were assessed by investigators per Response Evaluation Criteria in Solid Tumors v1.1. The denominator of BOR, ORR, and DCR is the number of patients with measurable disease and response evaluation

Abbreviations: BOR, best overall response; CI, confidence interval; CR, complete response; DCR, disease control rate; ORR, objective response rate; PD, progressive disease; PR, partial response; SD, stable disease

**Fig. 2:**
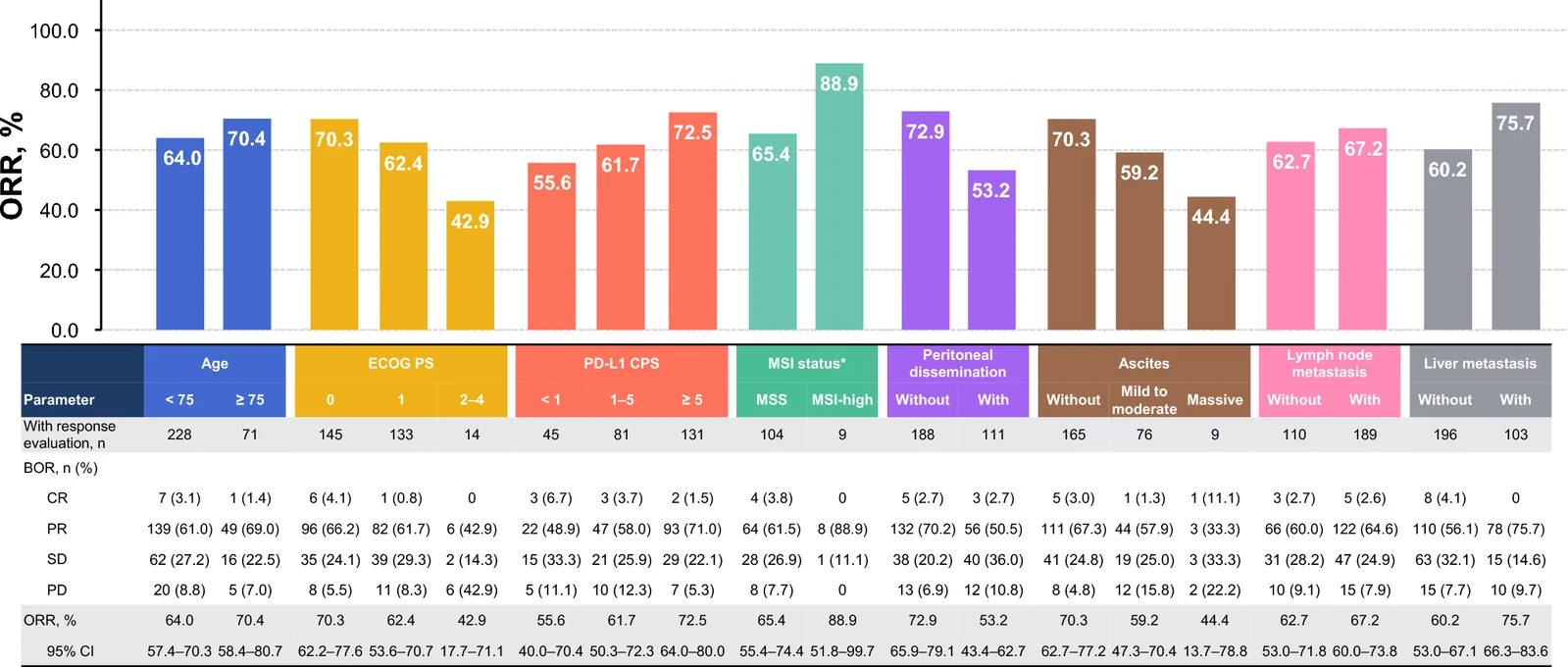
ORR by subgroup ORR was analyzed in patients with measurable disease and response evaluation, which were assessed by investigators per Response Evaluation Criteria in Solid Tumors v1.1. *MSI-low is not shown because only one patient was applicable. Abbreviations: BOR, best overall response; CI, confidence interval; CPS, combined positive score; CR, complete response; DCR, disease control rate; MSI, microsatellite instability; MSS, microsatellite stability; ORR, objective response rate; PD, progressive disease; PD-L1, programmed cell death ligand 1; PR, partial response; SD, stable disease

