# Supplementary material for: An observational study of the effectiveness and safety of nivolumab plus chemotherapy for untreated advanced or recurrent gastric cancer in Japanese real-world settings: the G-KNIGHT study
Source: Gastric Cancer. 2025 Jul 9;28(5):955–67. doi: 10.1007/s10120-025-01641-7 (PMC12378153; doi:10.1007/s10120-025-01641-7)
Supplement: Supplementary file 1 — Supplementary file1 (DOCX 292 KB) [file 10120_2025_1641_MOESM1_ESM.docx]

**Online Resources**

The authors have provided this appendix to give readers additional information about their work.

Supplement to: Shigenori Kadowaki, Tomoyuki Otsuka, Keiko Minashi, Shinichi Nishina, Hiroshi Yabusaki, Chiaki Inagaki, et al. An Observational Study of the Effectiveness and Safety of Nivolumab plus Chemotherapy for Untreated Advanced or Recurrent Gastric Cancer in Japanese Real-World Settings: Interim analysis of the G-KNIGHT study

**Table of Contents**

[Online Resource 1. List of investigators 3](#_Toc201661169)

[Online Resource 2. Study objectives 4](#_Toc201661170)

[Online Resource 3. Supplementary methods 5](#_Toc201661171)

[Online Resource 4. Patient diagram 6](#_Toc201661172)

[Online Resource 5. Patient characteristics by PD-L1 CPS and combination chemotherapy 7](#_Toc201661173)

[Online Resource 6. DOT by nivolumab plus chemotherapy regimen 8](#_Toc201661174)

[Online Resource 7. Details of second line treatment 9](#_Toc201661175)

[Online Resource 8. Association of patient characteristics and rwPFS 10](#_Toc201661176)

[Online Resource 9. Summary of safety by age and ECOG PS 11](#_Toc201661177)

[Online Resource 10. Time from nivolumab plus chemotherapy to onset of any grade irAEs 12](#_Toc201661178)

[Online Resource 11. Time from nivolumab plus chemotherapy to onset of grade ≥ 3 irAEs 13](#_Toc201661179)

[Online Resource 12. Time from onset to recovery of any grade irAEs 14](#_Toc201661180)

[Online Resource 13. Time from onset to recovery of grade ≥ 3 irAEs 15](#_Toc201661181)

Online Resource 1. List of investigators

| **Site name** | **Investigator name** |
| --- | --- |
| Aichi Cancer Center Hospital | Shigenori Kadowaki |
| Chiba Cancer Center | Keiko Minashi |
| Chiba University Hospital | Hisahiro Matsubara |
| Fujita Health University Hospital | Hiroshi Matsuoka |
| Fukui University Hospital | Takanori Goi |
| Gunma Prefectural Cancer Center | Hisashi Hosaka |
| Hyogo Cancer Center | Masahiro Tsuda |
| Kanagawa Cancer Center | Nozomu Machida |
| Kanazawa University Hospital | Noriyuki Inaki |
| Kindai University Hospital | Chiaki Inagaki |
| Kobe City Medical Center General Hospital | Hisateru Yasui |
| Kumamoto University Hospital | Satoshi Ida |
| Kurashiki Central Hospital | Shinichi Nishina |
| Kyorin University Hospital | Fumio Nagashima |
| Kyoto University Hospital | Junichi Matsubara |
| NHO Shikoku Cancer Center | Tomohiro Nishina |
| Niigata Cancer Center Hospital | Hiroshi Yabusaki |
| Osaka International Cancer Institute | Tomoyuki Otsuka |
| Osaka Red Cross Hospital | Takehiko Tsumura |
| Shiga General Hospital | Kazuyoshi Matsumura |
| St. Marianna University Hospital | Hiroyuki Arai |
| Tokai University Hospital | Masashi Matsushima |
| Wakayama Medical University Hospital | Takao Maekita |

All sites are Cancer Care Coordination Core Hospitals designated by the Ministry of Health, Labour and Welfare, established to provide high-quality cancer treatment throughout the country.

Online Resource 2. Study objectives

| Primary | - ORR - rwPFS - Incidence of irAE - Incidence of TRAE leading to treatment discontinuation |
| --- | --- |
| Secondary | - OS - DOT - DOR - DOT of second line treatment - rwPFS, ORR, and DOT of nivolumab in third or later line treatment - OS, rwPFS, ORR, DOT, and so on by patient characteristics - TNT - Proportion of patients received second line treatment - Safety by patient characteristics |
| Exploratory | - Degree of PD-L1 CPS - OS and rwPFS by duration of chemotherapy in nivolumab plus chemotherapy - Association of OS and treatment sequence |

Abbreviations: CPS, combined positive score; DOR, duration of response; DOT, duration of treatment; irAE, immune-related adverse event; ORR, objective response rate; OS, overall survival; PD-L1, programmed cell death ligand 1; rwPFS, real-world progression-free survival; TNT, time to next treatment; TRAE, treatment-related adverse event

Online Resource 3. Supplementary methods

Real-world progression-free survival was defined as the time from the date of nivolumab plus chemotherapy initiation to the date of the first documented progressive disease (PD) according to Response Evaluation Criteria in Solid Tumors (RECIST) v1.1, or death, whichever occurred first. Patients who underwent surgery after nivolumab plus chemotherapy were censored at the date of the surgery in the rwPFS analysis. Duration of response was defined as the time from the date of the first documented partial response or complete response after the nivolumab plus chemotherapy initiation to the date of the first documented PD or the date of death from any cause, whichever occurred first. Overall survival was defined as the time from the date of nivolumab plus chemotherapy initiation to the date of death from any cause. PD was determined not only by radiological progression based on RECIST v1.1, but also by clinical progression, which was assessed by worsening of disease status based on clinical symptoms, physical findings, tumor markers, and various test values. Time-to-next treatment was defined as the time from the date of nivolumab plus chemotherapy initiation to the date of second line treatment initiation or death, whichever occurred first. Duration of treatment was defined as the period from the initiation of first-line therapy to the physician-determined end date of treatment.


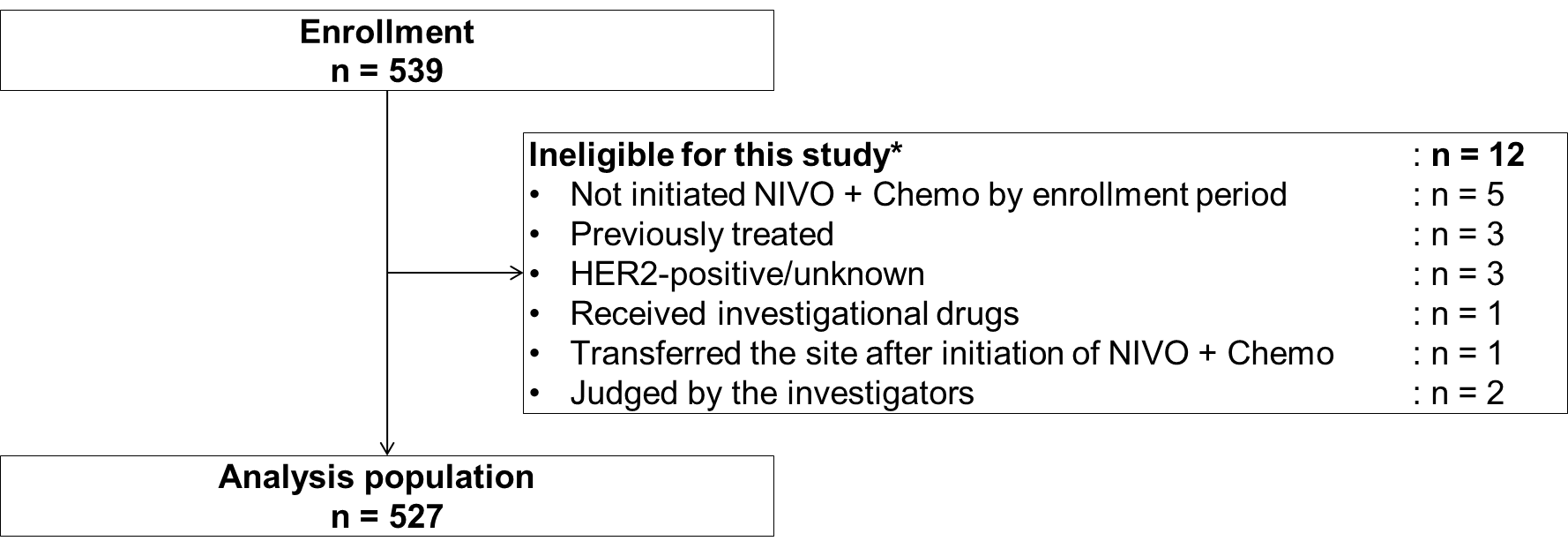


Online Resource 4. Patient diagram

* Some patients did not meet multiple criteria.
Abbreviations: HER2, human epidermal growth factor receptor 2; NIVO + Chemo, nivolumab plus chemotherapy.

Online Resource 5. Patient characteristics by PD-L1 CPS and combination chemotherapy

|  | PD-L1 CPS | | |  | Combination chemotherapy | | |
| --- | --- | --- | --- | --- | --- | --- | --- |
|  | < 1 | 1–5 | ≥ 5 |  | SOX | CapeOX | FOLFOX |
| Characteristics, n (%) | n = 89 | n = 143 | n = 216 |  | n = 387 | n = 34 | n = 106 |
| Age, years |  |  |  |  |  |  |  |
| Median (range) | 70.3 (34–85) | 70.2 (25–87) | 70.2 (25–87) |  | 70.4 (25-87) | 71.6 (51–82) | 68.8 (24–87) |
| < 65 | 27 (30.3) | 51 (35.7) | 71 (32.9) |  | 124 (32.0) | 8 (23.5) | 40 (37.7) |
| ≥ 65, < 75 | 46 (51.7) | 49 (34.3) | 90 (41.7) |  | 162 (41.9) | 16 (47.1) | 44 (41.5) |
| ≥ 75 | 16 (18.0) | 43 (30.1) | 55 (25.5) |  | 101 (26.1) | 10 (29.4) | 22 (20.8) |
| Male | 60 (67.4) | 93 (65.0) | 147 (68.1) |  | 256 (66.1) | 26 (76.5) | 63 (59.4) |
| ECOG PS |  |  |  |  |  |  |  |
| 0 | 45 (50.6) | 69 (48.3) | 103 (47.7) |  | 212 (54.8) | 18 (52.9) | 25 (23.6) |
| 1 | 33 (37.1) | 62 (43.4) | 98 (45.4) |  | 158 (40.8) | 15 (44.1) | 51 (48.1) |
| 2–4 | 9 (10.1) | 10 (7.0) | 12 (5.6) |  | 9 (2.3) | 0 | 27 (25.5) |
| Unknown | 2 (2.2) | 2 (1.4) | 3 (1.4) |  | 8 (2.1) | 1 (2.9) | 3 (2.8) |
| Disease status |  |  |  |  |  |  |  |
| Advanced | 59 (66.3) | 124 (86.7) | 192 (88.9) |  | 334 (86.3) | 14 (41.2) | 96 (90.6) |
| Recurrent or relapse | 30 (33.7) | 19 (13.3) | 24 (11.1) |  | 53 (13.7) | 20 (58.8) | 10 (9.4) |
| Metastasis |  |  |  |  |  |  |  |
| Liver | 12 (13.5) | 42 (29.3) | 61 (28.2) |  | 94 (24.2) | 10 (29.4) | 26 (24.5) |
| Peritoneal dissemination | 52 (58.4) | 69 (48.3) | 95 (44.0) |  | 170 (43.9) | 8 (23.5) | 65 (61.3) |
| Lymph node | 39 (43.8) | 84 (58.7) | 126 (58.3) |  | 214 (55.3) | 17 (50.0) | 60 (56.6) |
| No. of metastasis |  |  |  |  |  |  |  |
| 0 | 6 (6.7) | 7 (4.9) | 12 (5.6) |  | 32 (8.3) | 4 (11.8) | 4 (3.8) |
| 1 | 53 (59.6) | 69 (48.3) | 106 (49.1) |  | 198 (51.2) | 21 (61.8) | 42 (39.6) |
| ≥ 2 | 30 (33.7) | 67 (46.9) | 98 (45.4) |  | 157 (40.6) | 9 (26.5) | 60 (56.6) |
| Previous surgery | 38 (42.7) | 37 (25.9) | 43 (19.9) |  | 96 (24.8) | 24 (70.6) | 19 (17.9) |
| PD-L1 CPS IHC 28-8 |  |  |  |  |  |  |  |
| Tested | 89 (100) | 143 (100) | 216 (100) |  | 328 (84.8) | 28 (82.4) | 95 (89.6) |
| < 1 | 89 (100) | 0 | 0 |  | 63/328 (19.2) | 5/28 (17.9) | 21/95 (22.1) |
| 1–5 | 0 | 143 (100) | 0 |  | 102/328 (31.1) | 11/28 (39.3) | 30/95 (31.6) |
| ≥ 5 | 0 | 0 | 216 (100) |  | 163/328 (49.7) | 10/28 (35.7) | 43/95 (45.3) |
| Unclear | 0 | 0 | 0 |  | 0 | 2/28 (7.1) | 1/95 (1.1) |
| Untested | 0 | 0 | 0 |  | 29 (7.5) | 2 (5.9) | 6 (5.7) |
| Unknown | 0 | 0 | 0 |  | 30 (7.8) | 4 (11.8) | 5 (4.7) |
| MSI status |  |  |  |  |  |  |  |
| Tested | 50 (56.2) | 69 (48.3) | 79 (36.6) |  | 146 (37.7) | 15 (44.1) | 59 (55.7) |
| MSS | 43/50 (86.0) | 64/69 (92.8) | 66/79 (83.5) |  | 125/146 (85.6) | 14/15 (93.3) | 53/59 (89.8) |
| MSI-low | 0 | 0 | 3/79 (3.8) |  | 3/146 (2.1) | 0 | 0 |
| MSI-high | 1/50 (2.0) | 2/69 (2.9) | 8/79 (10.1) |  | 9/146 (6.2) | 0 | 4/59 (6.8) |
| Unclear | 6/50 (12.0) | 3/69 (4.3) | 2/79 (2.5) |  | 9/146 (6.2) | 1/15 (6.7) | 2/59 (3.4) |
| Untested | 21 (23.6) | 35 (24.5) | 84 (38.9) |  | 131 (33.9) | 6 (17.6) | 29 (27.4) |
| Unknown | 18 (20.2) | 39 (27.3) | 53 (24.5) |  | 110 (28.4) | 13 (38.2) | 18 (17.0) |
| Ascites |  |  |  |  |  |  |  |
| Without | 44 (49.4) | 72 (50.3) | 116 (53.7) |  | 215 (55.6) | 18 (52.9) | 38 (35.8) |
| With | 34 (38.2) | 58 (40.6) | 84 (38.9) |  | 135 (34.9) | 9 (26.5) | 60 (56.6) |
| Mild to moderate | 23/34 (67.6) | 51/58 (87.9) | 62/84 (73.8) |  | 106/135 (78.5) | 8/9 (88.9) | 43/60 (71.7) |
| Massive | 5/34 (14.7) | 4/58 (6.9) | 13/84 (15.5) |  | 13/135 (9.6) | 0 | 10/60 (16.7) |
| Unclear | 6/34 (17.6) | 3/58 (5.2) | 9/84 (10.7) |  | 16/135 (11.9) | 1/9 (11.1) | 7/60 (11.7) |
| Unknown | 11 (12.4) | 13 (9.1) | 16 (7.4) |  | 37 (9.6) | 7 (20.6) | 8 (7.5) |

Abbreviations: CapeOX, capecitabine/oxaliplatin; CPS, combined positive score; ECOG PS, Eastern Cooperative Oncology Group performance status; FOLFOX, 5-fluorouracil/leucovorin/oxaliplatin; IHC, immunohistochemistry; MSI, microsatellite instability; MSS, microsatellite stable; PD-L1, programmed cell death ligand 1; SOX, S-1/oxaliplatin.


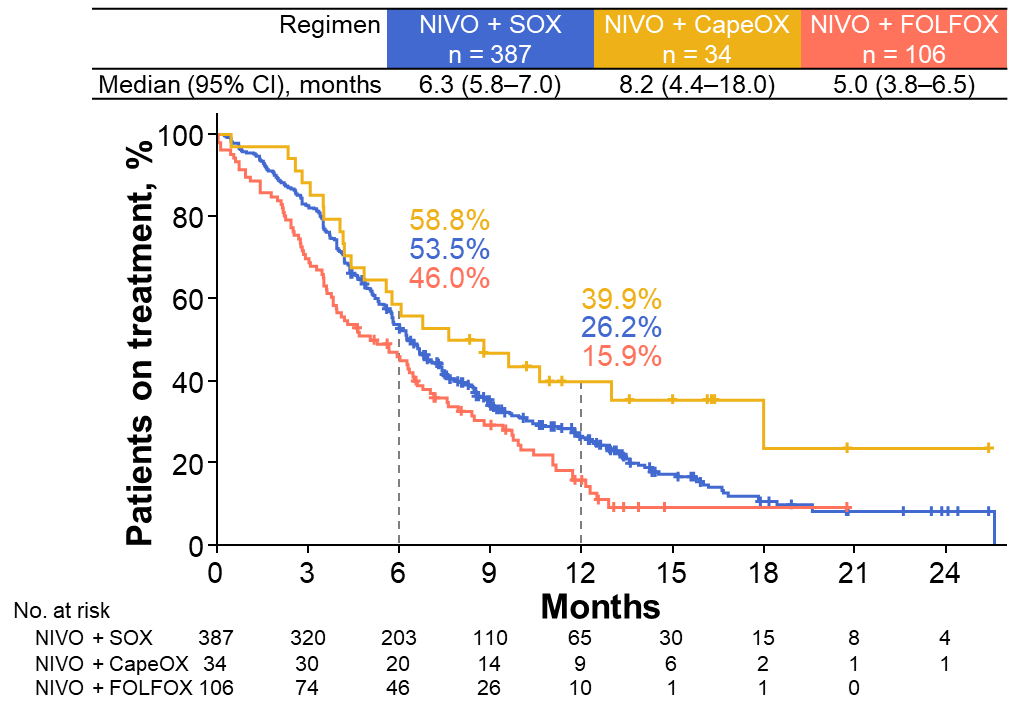


Online Resource 6. DOT by nivolumab plus chemotherapy regimen

DOT was defined as the period from the initiation of first-line therapy to the physician-determined end date of treatment.

A total of 11 patients were treated with multiple regimens, and they were categorized by the regimen with the longest duration of treatment in each patient.

Abbreviations: CapeOX, capecitabine/oxaliplatin; DOT, duration of treatment; FOLFOX, 5-fluorouracil/leucovorin/oxaliplatin; NIVO + Chemo, nivolumab plus chemotherapy; SOX, S-1/oxaliplatin.

Online Resource 7. Details of second line treatment

| Parameters | Overall  n = 196/413 (47.5)* |
| --- | --- |
| Time to next line treatment, months, median (range) | 6.7 (0.7–21.0) |
| Second line treatment regimen, n (%) |  |
| Ramucirumab plus paclitaxel^†^ | 79 (40.3) |
| Ramucirumab plus nab-paclitaxel^†^ | 79 (40.3) |
| nab-paclitaxel | 9 (4.6) |
| Ramucirumab plus irinotecan | 4 (2.0) |
| Docetaxel | 2 (1.0) |
| Irinotecan | 1 (0.5) |
| Ramucirumab | 3 (1.5) |
| Pembrolizumab | 1 (0.5) |
| Others | 19 (9.7) |
| Treatment status, n (%) |  |
| On-going | 56 (28.6) |
| Discontinued | 140 (71.4) |
| Discontinued reason, n (%) |  |
| Ineffective | 100 (51.0) |
| Adverse event | 18 (9.2) |
| Patient requests | 5 (2.6) |
| Death | 4 (2.0) |
| Other reasons | 12 (6.1) |
| Unknown | 1 (0.5) |

* A total of 114 patients who were still receiving nivolumab plus chemotherapy were excluded from total population. ^†^ One patient received both paclitaxel plus ramucirumab and nab-paclitaxel plus ramucirumab as second-line therapy.

Online Resource 8. Association of patient characteristics and rwPFS

|  |  |  | PD or death*, n (%) | | Univariable analysis | |  | Multivariable analysis | |
| --- | --- | --- | --- | --- | --- | --- | --- | --- | --- |
| Subgroup |  | n* | Without | With | HR (95% CI) | P-value |  | Adjusted HR (95% CI) | P-value |
| Sex | Male | 344 | 120 (34.9) | 224 (65.1) |  | 0.268 |  |  | 0.537 |
|  | Female | 182 | 55 (30.2) | 127 (69.8) | 1.13 (0.91–1.41) |  |  | 1.09 (0.82–1.46) |  |
| Age | < 75 years | 393 | 129 (32.8) | 264 (67.2) |  | 0.629 |  |  | 0.511 |
|  | ≥ 75 years | 133 | 46 (34.6) | 87 (65.4) | 0.94 (0.74–1.20) |  |  | 1.11 (0.82–1.49) |  |
| ECOG PS | 0–1 | 478 | 167 (34.9) | 311 (65.1) |  | <0.001 |  |  | 0.006 |
|  | 2–4 | 36 | 5 (13.9) | 31 (86.1) | 2.84 (1.96–4.12) |  |  | 2.04 (1.23–3.38) |  |
| PD-L1 CPS | < 1 | 88 | 28 (31.8) | 60 (68.2) |  | 0.754 |  |  | 0.222 |
|  | 1–5 | 143 | 49 (34.3) | 94 (65.7) | 1.12 (0.81–1.55) |  |  | 1.17 (0.79–1.73) |  |
|  | ≥ 5 | 216 | 76 (35.2) | 140 (64.8) | 1.03 (0.76–1.39) |  |  | 0.90 (0.62–1.30) |  |
| Surgical history for gastric cancer | Without | 388 | 115 (29.6) | 273 (70.4) |  | 0.038 |  |  | 0.991 |
|  | With | 138 | 60 (43.5) | 78 (56.5) | 0.77 (0.59–0.99) |  |  | 1.00 (0.71–1.41) |  |
| Peritoneal dissemination metastasis | Without | 283 | 100 (35.3) | 183 (64.7) |  | 0.848 |  |  |  |
|  | With | 243 | 75 (30.9) | 168 (69.1) | 1.02 (0.83–1.26) |  |  |  |  |
| Number of metastatic sites | ≤ 1 | 260 | 95 (36.5) | 165 (63.5) |  | 0.013 |  |  | 0.167 |
|  | ≥ 2 | 226 | 62 (27.4) | 164 (72.6) | 1.32 (1.06–1.63) |  |  | 1.24 (0.92–1.67) |  |
| Liver metastasis | Without | 396 | 141 (35.6) | 255 (64.4) |  | 0.042 |  |  | 0.498 |
|  | With | 130 | 34 (26.2) | 96 (73.8) | 1.28 (1.01–1.62) |  |  | 1.13 (0.79–1.63) |  |
| Disease status | Advanced | 443 | 145 (32.7) | 298 (67.3) |  | 0.038 |  |  |  |
|  | Recurrent or relapse | 83 | 30 (36.1) | 53 (63.9) | 0.73 (0.54–0.98) |  |  |  |  |
| Ascites | Without | 270 | 113 (41.9) | 157 (58.1) |  | <0.001 |  |  | 0.001 |
|  | Mild to moderate | 157 | 33 (21.0) | 124 (79.0) | 1.69 (1.33–2.14) |  |  | 1.26 (0.94–1.70) |  |
|  | Massive | 23 | 3 (13.0) | 20 (87.0) | 3.07 (1.92–4.91) |  |  | 2.79 (1.60–4.86) |  |
| Albumin | Per 1 g/dL increase | 520 | 174 (33.5) | 346 (66.5) | 0.65 (0.55–0.77) | <0.001 |  | 0.76 (0.61–0.96) | 0.020 |
| Lactate dehydrogenase | Per 1 U/L increase | 516 | 173 (33.5) | 343 (66.5) | 1.00 (1.00–1.00) | 0.003 |  | 1.00 (1.00–1.00) | 0.382 |
| Glasgow prognostic score^†^ | 0 | 254 | 111 (43.7) | 143 (56.3) |  | <0.001 |  |  |  |
|  | 1 | 208 | 53 (25.5) | 155 (74.5) | 1.43 (1.14–1.79) |  |  |  |  |
|  | 2 | 48 | 8 (16.7) | 40 (83.3) | 2.40 (1.69–3.41) |  |  |  |  |
| C-reactive protein | Per 1 mg/dL increase | 510 | 172 (33.7) | 338 (66.3) | 1.09 (1.05–1.13) | <0.001 |  |  |  |
| White blood cell | Per 1 count/mm^3^ increase | 519 | 174 (33.5) | 345 (66.5) | 1.00 (1.00–1.00) | <0.001 |  |  |  |
| Hemoglobin | Per 1 g/dL increase | 519 | 174 (33.5) | 345 (66.5) | 0.93 (0.88–0.98) | 0.010 |  |  |  |
| Neutrophil | Per 1 count/mm^3^ increase | 350 | 122 (34.9) | 228 (65.1) | 1.00 (1.00–1.00) | 0.001 |  |  |  |
| Alkaline phosphatase | Per 10 U/L increase | 520 | 174 (33.5) | 346 (66.5) | 1.02 (1.01–1.02) | <0.001 |  |  |  |

Variables included in the multivariable analysis were selected according to their clinical relevance, statistical significance in the univariable analysis (cutoff, p < 0.05), and correlation between variables.

* The number of patients indicates those included in the univariable analysis.

^†^ Patients who had both a serum C-reactive protein > 1.0 mg/dL and albumin < 3.5 g/dL were allocated a GPS of 2. Patients with only one of the abnormal values were allocated a GPS of 1, and patients who had neither been allocated a GPS of 0.

Abbreviations: CI, confidence interval; CPS, combined positive score; ECOG PS, Eastern Cooperative Oncology Group performance status; HR, hazard ratio; PD, progressive disease; PD-L1, programmed cell death ligand 1; rwPFS, real-world progression-free survival.

Online Resource 9. Summary of safety by age and ECOG PS

|  | Age | |  | ECOG PS* | | |
| --- | --- | --- | --- | --- | --- | --- |
|  | < 75 years | ≥ 75 years |  | 0 | 1 | 2–4 |
| Category | n = 394 | n = 133 |  | n = 255 | n = 224 | n = 36 |
| Any grade TRAE, n (%) | 361 (91.6) | 120 (90.2) |  | 239 (93.7) | 204 (91.1) | 28 (77.8) |
| Grade 3–4 TRAE, n (%) | 171 (43.4) | 42 (31.6) |  | 102 (40.0) | 88 (39.3) | 19 (52.8) |
| TRAE leading to discontinuation, n (%) | 37 (9.4) | 18 (13.5) |  | 23 (9.0) | 28 (12.5) | 2 (5.6) |
| Any grade irAE, n (%) | 102 (25.9) | 34 (25.6) |  | 73 (28.6) | 53 (23.7) | 6 (16.7) |
| Grade 3–4 irAE, n (%) | 35 (8.9) | 6 (4.5) |  | 18 (7.1) | 21 (9.4) | 1 (2.8) |
| Treatment-related death | 3 (0.8) | 2 (1.5) |  | 1 (0.4) | 3 (1.3) | 0 |

The severity of AEs was assessed according to Common Terminology Criteria for Adverse Events v5.0 by the investigators. AE information, such as whether the event was an irAE, its severity, and its causal relationship with the treatment, and its outcome, was assessed by the investigators.

* ECOG PS was unknown for a total of 12 patients.

Abbreviations: AE, adverse event; ECOG PS, Eastern Cooperative Oncology Group performance status; irAE, immune-related adverse event; TRAE, treatment-related adverse event.

**
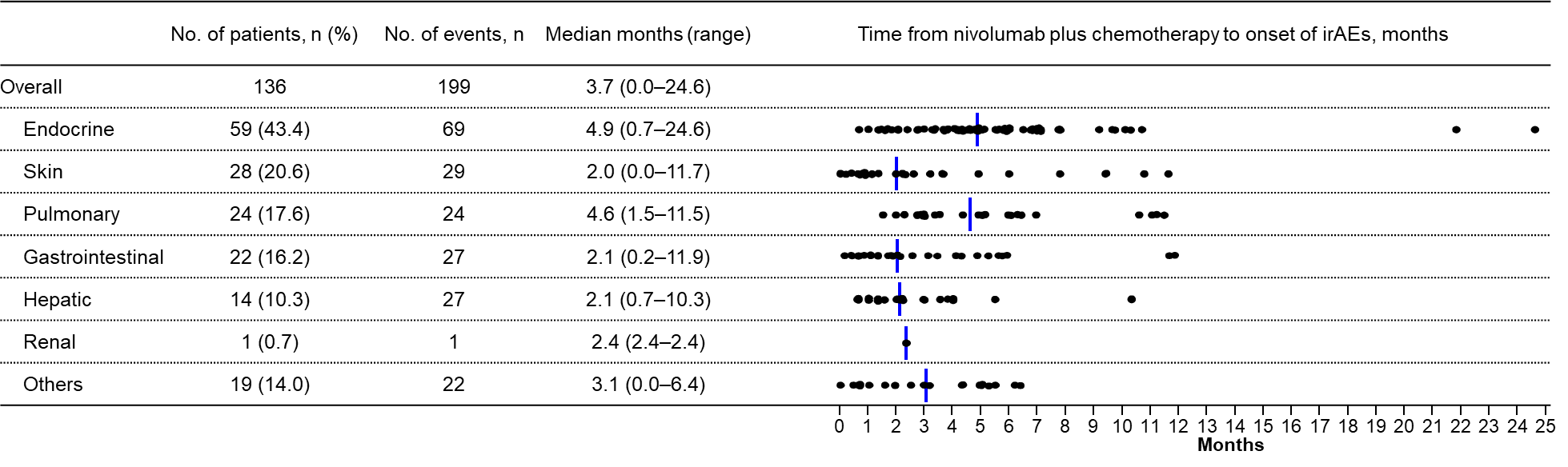
**

Online Resource 10. Time from nivolumab plus chemotherapy to onset of any grade irAEs

Blue vertical lines represent the median value. The severity of AEs was assessed according to Common Terminology Criteria for Adverse Events v5.0 by the investigators. AE information, such as whether the event was an irAE, its severity, and its causal relationship with the treatment, and its outcome, was assessed by the investigators.

Abbreviation: AE, adverse event; irAE, immune-related adverse event


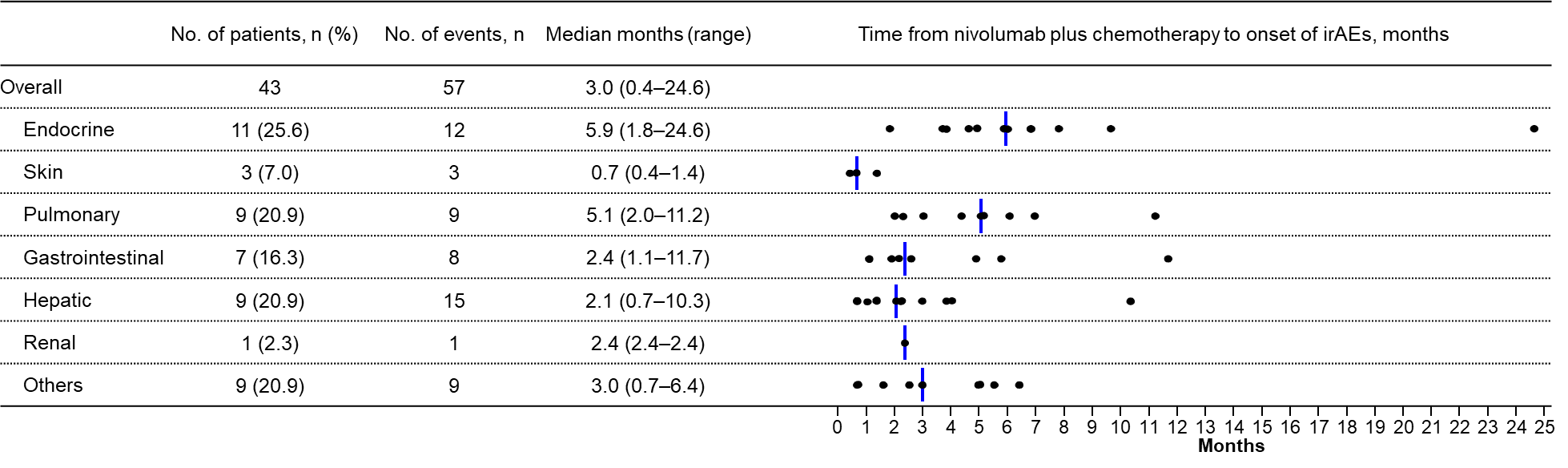


Online Resource 11. Time from nivolumab plus chemotherapy to onset of grade ≥ 3 irAEs

Blue vertical lines represent the median value. The severity of AEs was assessed according to Common Terminology Criteria for Adverse Events v5.0 by the investigators. AE information, such as whether the event was an irAE, its severity, and its causal relationship with the treatment, and its outcome, was assessed by the investigators.

Abbreviation: AE, adverse event; irAE, immune-related adverse event


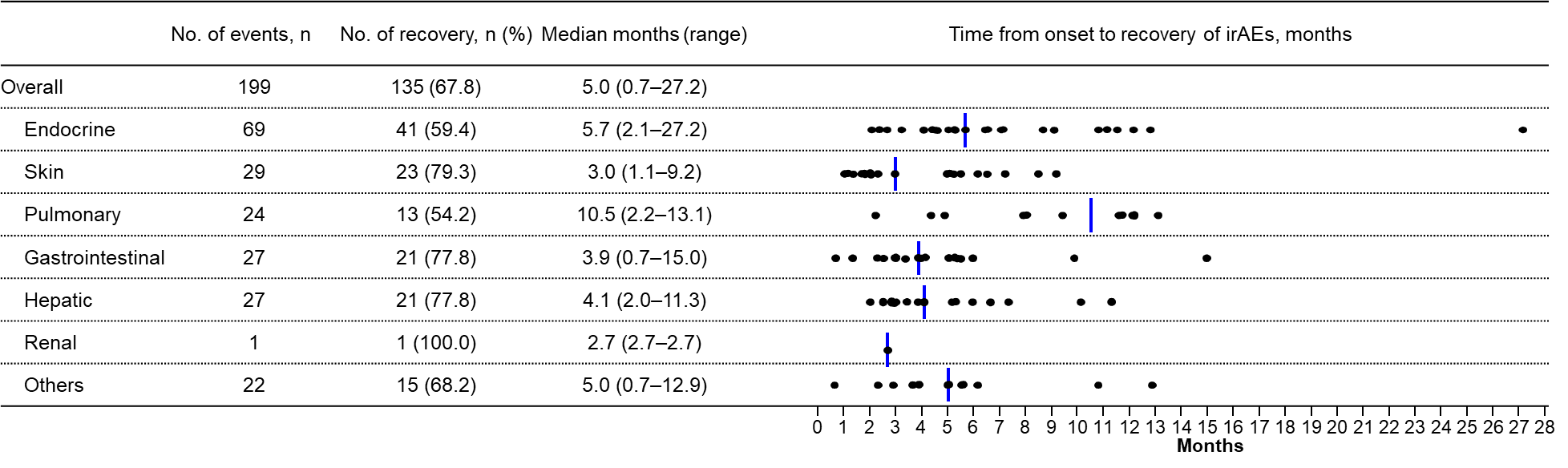


Online Resource 12. Time from onset to recovery of any grade irAEs

Blue vertical lines represent the median value. The severity of AEs was assessed according to Common Terminology Criteria for Adverse Events v5.0 by the investigators. AE information, such as whether the event was an irAE, its severity, its causal relationship to treatment, and its outcome, was analyzed according to investigator assessment. Recovery included recovered, recovering, and recovered but with sequelae.

Abbreviation: AE, adverse event; irAE, immune-related adverse event


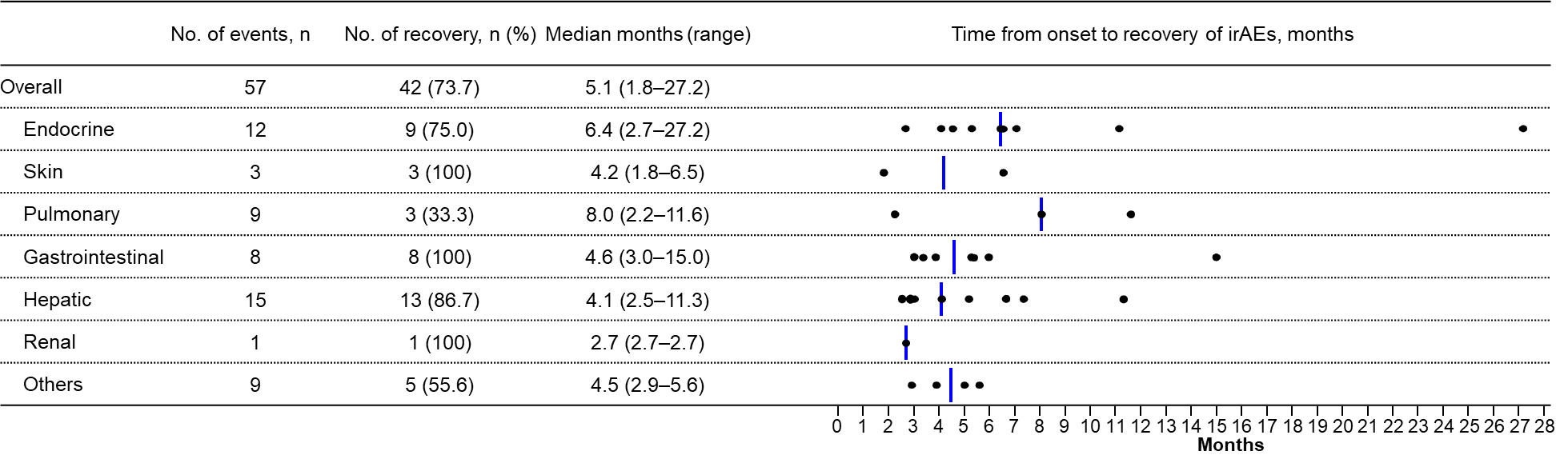


Online Resource 13. Time from onset to recovery of grade ≥ 3 irAEs

Blue vertical lines represent the median value. The severity of AEs was assessed according to Common Terminology Criteria for Adverse Events v5.0 by the investigators. AE information, such as whether the event was an irAE, its severity, its causal relationship to treatment, and its outcome, was analyzed according to investigator assessment. Recovery included recovered, recovering, and recovered but with sequelae.

Abbreviation: AE, adverse event; irAE, immune-related adverse event
